# Does Eating Chicken Feet With Pickled Peppers Cause Avian Influenza? Observational Case Study on Chinese Social Media During the Avian Influenza A (H7N9) Outbreak

**DOI:** 10.2196/publichealth.8198

**Published:** 2018-03-29

**Authors:** Bin Chen, Jian Shao, Kui Liu, Gaofeng Cai, Zhenggang Jiang, Yuru Huang, Hua Gu, Jianmin Jiang

**Affiliations:** ^1^ Zhejiang Provincial Center for Disease Control and Prevention Hangzhou China; ^2^ College of Computer Science, Zhejiang University Hangzhou China; ^3^ Johns Hopkins Bloomberg School of Public Health Baltimore, MD United States

**Keywords:** social media, misinformation, infodemiology, avian influenza A, disease outbreak

## Abstract

**Background:**

A hot topic on the relationship between a popular avian-origin food and avian influenza occurred on social media during the outbreak of the emerging avian influenza A (H7N9). The misinformation generated from this topic had caused great confusion and public concern.

**Objective:**

Our goals were to analyze the trend and contents of the relevant posts during the outbreak. We also aimed to understand the characteristics of the misinformation and to provide suggestions to reduce public misconception on social media during the emerging disease outbreak.

**Methods:**

The original microblog posts were collected from China’s Sina Weibo and Tencent Weibo using a combination of keywords between April 1, 2013 and June 2, 2013. We analyzed the weekly and daily trend of the relevant posts. Content analyses were applied to categorize the posts into 4 types with unified sorting criteria. The posts’ characteristics and geographic locations were also analyzed in each category. We conducted further analysis on the top 5 most popular misleading posts.

**Results:**

A total of 1680 original microblog posts on the topic were retrieved and 341 (20.30%) of these posts were categorized as misleading messages. The number of relevant posts had not increased much during the first 2 weeks but rose to a high level in the next 2 weeks after the sudden increase in number of reported cases at the beginning of week 3. The posts under “misleading messages” occurred and increased from the beginning of week 3, but their daily posting number decreased when the daily number of posts under “refuting messages” outnumbered them. The microbloggers of the misleading posts had the lowest mean rank of followers and previous posts, but their posts had a highest mean rank of posts. The proportion of “misleading messages” in places with no reported cases was significantly higher than that in the epidemic areas (23.6% vs 13.8%). The popular misleading posts appeared to be short and consisted of personal narratives, which were easily disseminated on social media.

**Conclusions:**

Our findings suggested the importance of responding to common questions and misconceptions on social media platforms from the beginning of disease outbreaks. Authorities need to release clear and reliable information related to the popular topics early on. The microbloggers posting correct information should be empowered and their posts could be promoted to clarify false information. Equal importance should be attached to clarify misinformation in both the outbreak and nonoutbreak areas.

## Introduction

A novel avian influenza A (H7N9) case was first reported in China on March 31, 2013 [1]. A total of 131 confirmed cases and 39 deaths were documented in China as of May 31, 2013 [2]. From May, the first outbreak subsided, with only 1 case reported each month in June and July [3,4]. Due to its severity, this outbreak drew great public attention from all over the world [5].

Emerging infectious disease outbreaks usually drive people to search for more relevant information because of the uncertainty about the source of infection and fear of the deadly consequences [6,7]. In China, Sina Weibo and Tencent Weibo were the two largest social media platforms with more than 300 million users when the outbreak occurred [8]. Weibo is the Chinese word for microblog and allows a 140-character limit. The Internet users who post on “Weibo” are known as microbloggers. Driven by the popularity of such platforms, the outbreak triggered great public concern, with over 850,000 posts related to the term of “H7N9” generated in Sina Weibo [5]. With the function of self-expression and networking, social media provides a fertile ground for unreliable information generation and circulation [6,9,10]. One study showed that 57% of the rumors about the tragedy of Malaysia Airlines MH370 were generated from social media platforms in China [11]. Such misinformation or rumors could lead to great confusion and pose challenges to the disease control [7,12]. The outbreak of severe acute respiratory syndrome in 2003 serves as a typical example where rumors stirred great panic in Chinese society and made it hard for disease prevention and control [7,13].

From the beginning of the outbreak, social media users started to query the safety of the avian-origin food. Due to the poultry origin, a kind of Chinese traditional food—Chicken feet with pickled pepper—sparked microbloggers’ discussion about its causal relationship with the avian influenza. Chicken feet with pickled pepper (known as Pao-jiao-feng-zhua in Chinese) is a popular food item widely loved by Chinese people. The chicken feet are boiled for about 20 min and pickled with peppers for 2 days or longer. Although the avian-origin influenza A (H7N9) virus exists in the live poultry, the virus is sensitive to heat and cannot survive in the boiling water for 2 min [14,15]. Therefore, the virus has no chance of survival during the production process of the chicken feet with pepper. However, the topic on the relationship between the 2 items was widely discussed. Many microbloggers released messages that misunderstood the causal relationship between the 2 counterparts, and their posts became very prevalent [16-18]. The false messages claiming that chicken feet with pepper would cause H7N9 influenza were identified as rumors and caused great confusion and panic during the outbreak [17].

Little research has been done to examine the information and misinformation generated on social media during emerging disease outbreaks in China. The purpose of this observational case study is to understand the trend and characteristics of the relevant Weibo posts on the hot topic and to give the recommendations for effective health communication practice on social media in the context of emerging disease outbreaks.

## Methods

### Data Collection

We used a combination of *H7N9* or *Qinliugan* (the Chinese equivalent for avian influenza) and “Pao-jiao-feng-zhua” (the Chinese equivalent for chicken feet with pickled peppers) as keywords to search for the relevant original microblog posts from Sina Weibo and Tencent Weibo in September 2013. An original microblog post is defined as a short post where the content was not copied or forwarded from the news media or somewhere else [16,19]. The selected study period spanned 63 days or 9 weeks between April 1, 2013 (1 day after the official report of the first cases) and June 2, 2013 (when the government began to report the cases monthly instead of daily and weekly, indicating the outbreak was basically under control). We recorded the content of the posts, along with the posting date, geographic location, replies, number of retweets, number of microbloggers’ followers, and number of microbloggers’ existing posts. We examined the posting trend by weeks based on the posting date. The weekly reported case numbers were collected from the mainstream media.

### Content Analysis

About 11.90% (200/1680) were randomly selected from the collected posts and reviewed by a panel of 3 researchers. Each researcher independently read the contents of each post and synthesized different themes. After that, the panel discussed all generated themes and made an agreement to code all the posts (1680) into 4 major categories: queries, misleading messages, refuting messages, and other messages. We set the sorting criteria before the coding process. Queries referred to the posts querying whether eating chicken feet with pickled peppers caused avian influenza A (H7N9). If the microbloggers asked rhetorical questions or the question was answered by themselves in the same posts, such posts were not included in this category. Misleading messages referred to posts claiming that influenza A (H7N9) was caused by chicken feet with pickled peppers or to the posts persuading other people to believe the incorrect statement. Refuting messages included posts that stated that there was no causal relationship between the 2 counterparts and posts that corrected the false information or reminded the readers not to believe the wrong messages. Other messages represented posts that could not be classified into the categories above. The posts in this category have not mentioned the relationship between eating chicken feet with pickled pepper and H7N9 avian influenza, or the information is too limited to be judged.

**Table 1:** The coding criteria and the sample posts.

Categories	Definition	Sample posts
Queries	Posts querying whether eating chicken feet with pickled peppers caused avian influenza A (H7N9). Posts with rhetorical questions by microbloggers or answering of the question by themselves in the same posts were not included.	With so many H7N9 cases, is it dangerous or safe to eat chicken feet with pickled peppers? I ate chicken feet with pickled peppers last night. Am I going to have avian influenza? Is it the truth that the new H7N9 cases were caused by chicken feet with pickled peppers in our city?
Misleading messages	Posts claiming that influenza A (H7N9) was caused by chicken feet with pickled peppers or posts persuading other people to believe the incorrect statement.	Our city had the first case of avian influenza caused by chicken feet with pickled peppers last night! Don’t eat chicken feet with pickled peppers because it causes H7N9. Tell the people around you. A staff of my company was infected by H7N9 virus just because he had chicken feet with pickled peppers last night.
Refuting messages	Posts which stated that there was no causal relationship between the 2 counterparts and posts which corrected the false information or reminded the readers not to believe the wrong messages.	Chicken feet with pickled peppers have no chance to carry H7N9 virus and cause illnesses. It’s really silly to believe that chicken feet with pickled peppers would cause H7N9 flu. Don’t spread the false message (Chicken feet with pickled peppers cause H7N9 avian influenza) among the public.
Other messages	Posts that could not be classified into the categories above. The posts in this category have not mentioned the relationship between eating chicken feet with pickled pepper and H7N9 infection, or the information is too limited.	Chicken feet with pickled peppers is hot but H7N9 avian influenza is cold. Avian influenza and my loved chicken feet with pickled peppers…

Two researchers of the same panel independently reviewed all the 1680 posts and categorized them based on the sorting criteria. The coefficient of agreement (Cohen kappa) of the coding results between the 2 reviewers was .82. The third reviewer discussed with the 2 reviewers about the posts with different coding results and finalized the categorization. Some sample posts are listed in Table 1.

### Statistical Analysis

We used WPS Office Excel (Kingsoft Office Software, Beijing, China) to plot the weekly trend of posting. The weekly and daily posts were presented in bar charts by the categories we defined earlier. Kruskal Wallis tests were performed to analyze the characteristics and potential impact of the posts in each category. We also used Pearson chi-square test to compare geographic locations of the posts by category. We further examined the popular “Misleading messages” with regard to their features. A *P* value <.05 was considered statistically significant.

The study has been reviewed and approved by the Institutional Ethics Committee in the Zhejiang Provincial Centers for Disease Control and Prevention. The identity of the microbloggers was kept confidential.

## Results

A total of 1680 original microblog posts containing “chicken feet with pickled peppers” and “H7N9” or “avian influenza” were retrieved from the 2 largest microblog platforms, with 1353 posts (80.54%) from Sina Weibo and 327 posts (19.46%) from Tencent Weibo.

### Posting Trend of Posts Related to the Topic

**Figure 1:**
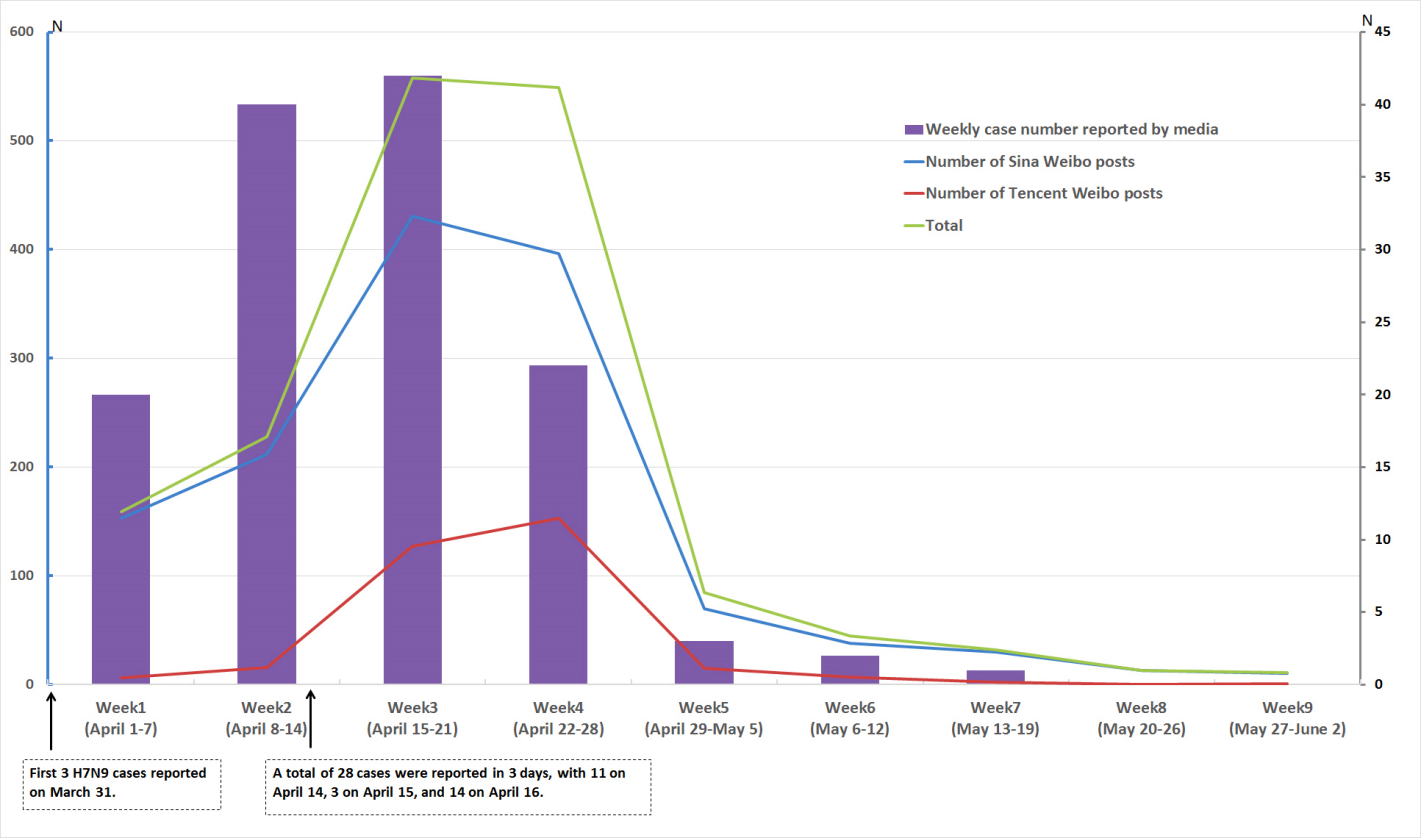
Weekly posting trend of the observed microblog posts related to the topic and the number of weekly reported cases from April 1 to June 2, 2013.

The weekly posting trends were similar between Sina Weibo and Tencent Weibo for our topic, except in week 4. The posting trend was consistent with the weekly reporting trend of H7N9 avian influenza case number. The posts increased gradually in the first 2 weeks (April 1-14) after the official announcement of the first 3 H7N9 cases in the Shanghai municipality and Anhui Province of China on March 31. From March 31 to April 13, the number of daily reported cases was ≤6. However, 28 new cases were reported in just 3 days, spanning from April 14 to April 16, with 11 new cases on April 14 and 14 cases on April 16. Subsequently, the weekly posts rose sharply from the start of week 3 (April 15-21) and peaked at 558 and remained high (549) in week 4 (April 22-28), which corresponded to more than 1100 posts in 2 weeks (April 15-28). In week 5, the posts dropped substantially and then gradually declined to a small number during weeks 6 to 9 (Figure 1).

### Contents of the Posts and the Posting Trend of Posts Under Different Categories

Among the 1680 posts, 466 (27.74%) posts were classified as queries, 341 (20.30%) as misleading messages, 508 (30.24%) as refuting messages, and 365 (21.73%) as other messages.

**Figure 2:**
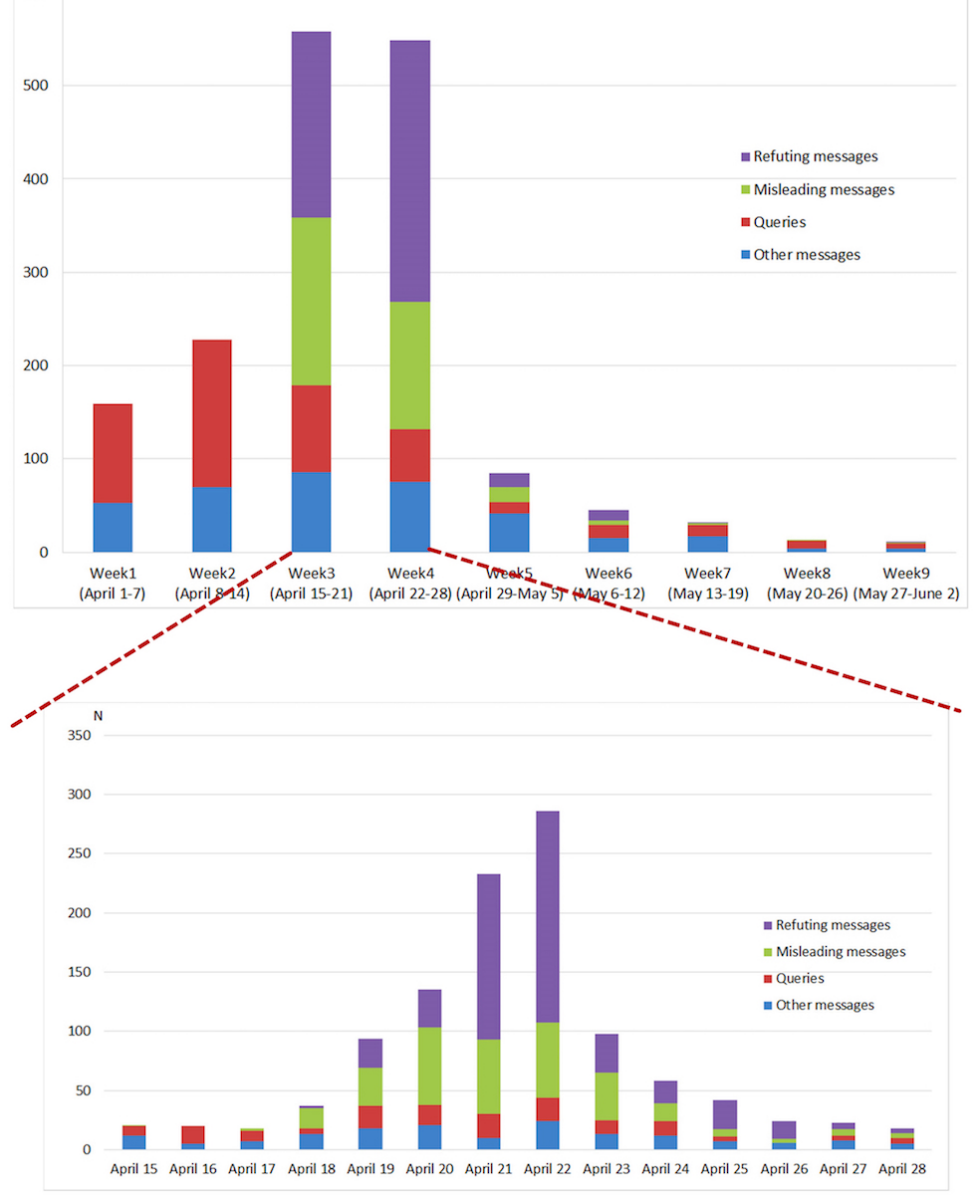
Weekly and daily microblog posts, by categoriescategory, during the periods from April 1 to June 2 and April 15 to April 28, 2013.

Most of the posts dated in the first 2 weeks were under queries, with 66.7% (106/159) and 69.3% (158/228) in the first and second weeks, respectively. There were 33.3% (53/159) and 30.7% (70/228) posts recognized as other messages in the first 2 weeks. No posts under misleading messages and refuting messages were identified in the same period. In the third week, the posts under misleading messages increased to 32.3% (180/558) per week, yet the posts under refuting messages also appeared and rose to 35.7% (199/558) per week. The number of posts under “queries” decreased to 16.7% (93/558) and other messages kept steady at 15.4% (86/558). “Misleading messages” reduced to 24.7% (136/549) in the fourth week, whereas the number of posts under refuting messages peaked at 51.2% (281/549). The posts under the other 2 categories showed a steady trend in week 4. From week 5 to week 9, the posts of all 4 categories showed a downward trend, and the weekly posts dropped below 100 (Figure 2).

We took a closer look into the daily posting trend between week 3 and week 4 when most of the posts occurred. The first misleading message was identified on April 15. Three days later (April 18), the number of daily posts became 17 per day and increased to 32 on April 19. From April 20 to April 22, the number of “misleading messages” was kept at high levels of 65, 63, and 63 per day, respectively. Along with the rising “misleading” posts, the number of refuting messages started to increase from April 18 and peaked at 179 on April 22. The posts under “misleading messages” outnumbered those under refuting messages before April 20, but a reverse was observed in the next 2 days because of a sharp growth of the posts under refuting messages. The other 2 kinds of posts showed a relatively steady trend during the above-mentioned period. In the weeks from April 22, the posts of all 4 categories gradually dropped to a low level on April 28 (Figure 2).

### Characteristics of the Posts Under Different Categories

The characteristics of the posts under the 4 categories were different. The group of posts under misleading messages had the highest mean ranks of the number of retweets among the 4 groups (*P*<.001). However, the microbloggers of the posts under misleading messages had the lowest mean rank in terms of the numbers of followers and existing posts (*P*<.001). Their misleading posts had modest number of replies (mean rank=793.8). The posts under refuting messages were different in that their microbloggers had more followers and previous posts, but they had the lowest mean rank of the number of retweets (*P*<.001). The refuting messages received most comments among the 4 groups (*P*<.001). The posts under “queries” and “other messages” had moderate mean ranks of the number of microbloggers’ followers and previous post numbers (Table 2).

**Table 2:** Characteristics of the posts and their microbloggers under different categories.

Categories	Queries (N=466)	Misleading messages (N=341)	Refuting messages (N=508)	Other messages (N=365)	Chi-square (degrees of freedom)	*P* value
Mean rank of retweets	762.8	1050.1	852.3	727.4	137.1 (3)	<.001
Mean rank of comments	907.1	793.8	696.6	999.5	124.2 (3)	<.001
Mean rank of microbloggers’ follower number	875.3	637.7	971.7	971.7	101.3 (3)	<.001
Mean rank of microbloggers’ previous post number	843.7	651.0	968.1	835.9	87.3 (3)	<.001

**Table 3:** Geographic locations of the posts by category. Pearson χ^2^_3_=36.7, *P*<.001.

Geographic location of microbloggers’ ID	Queries, n (%)	Misleading messages, n (%)	Refuting messages, n (%)	Other messages, n (%)
Provinces with avian influenza A (H7N9) cases reported	151 (26.4)	79 (13.8)	191 (33.3)	152 (26.5)
Provinces or places with no cases reported (including countries abroad)	249 (28.8)	204 (23.6)	264 (30.5)	149 (17.2)
Total	400 (27.8)	283 (19.7)	455 (31.6)	301 (20.9)

**Top 5 most popular misleading posts.**

Top 5 posts in terms of the number of replies and forwards

- Shenzhen municipality confirmed the first H7N9 cases at 2:25 am. The chicken feet with pickled peppers the patient ate had a lot of virus. Please boil them before eating.
- I have heard people got H7N9 avian influenza because of chicken feet with pickled pepper. Anyway, ban chicken feet with pickled pepper for our life!
- Xiamen municipality reported an H7N9 case. The virus infected people through chicken feet with pickled pepper…
- I heard eating chicken feet with pickled pepper would cause H7N9. I have just ate one pack. Be careful, my friend should.
- The first H7N9 case was reported in our city. The patient ate a lot of chicken feet with pickled pepper. Don’t eat this food!

Top 5 posts released by microbloggers with most followers

- Shenzhen municipality has reported an H7N9 case, and the cause of disease was that the patient ate chicken feet with pickled pepper which carried a lot of H7N9 viruses. I request all my friends not to eat chicken feet with pickled pepper!
- One of my classmates ate chicken feet with pickled pepper and is dying with avian influenza A (H7N9).
- Don’t eat chicken feet with pickled pepper because H7N9 virus has been confirmed in that food.
- Luoyang municipality had the first H7N9 case and the transmission route was eating chicken feet with pickled pepper.
- Taiyuan municipality reported an avian influenza case. Examination of the patient’s food discovered that the chicken feet with pickled pepper had a lot of viruses!

### Geographic Distribution of the Misleading Messages and the Contents of the Top 5 Most Popular Misleading Posts

Geographic locations (IDs) of 1439 microblog posts were obtained, among which 573 posts were located in the provinces where avian influenza A (H7N9) cases were reported, and 866 posts were from the provinces or regions where no cases were reported. The proportion of rumor posts in the places with no reported cases was significantly higher than that in the epidemic areas (23.6%>13.8%; Table 3).

The top 5 posts under misleading messages in terms of the number of retweets and replies are listed in Textbox 1. Three of them claimed that H7N9 avian influenza cases were reported in the local areas, and the cause was eating chicken feet with pickled peppers. The other 2 posts expressed the microbloggers’ concern, stating that the microbloggers had heard of people getting avian influenza after eating chicken feet with pickled peppers and reminded the readers not to eat such food. Of the top 5 misleading posts with most followers, 3 claimed that their local area had H7N9 cases due to eating chicken feet with pickled peppers, one claimed that his/her classmate was dying because he/she had eaten chicken feet with pickled peppers, and the other suggested not eating chicken feet with pickled peppers to prevent infection with avian influenza A (H7N9) virus (Textbox 1).

## Discussion

### Principal Findings

Using the keyword search, we collected all the original posts relevant to the hot topic on *chicken feet with pickled peppers* and *H7N9* or *avian influenza* in the study period. Employing methods provided by digital epidemiology, we mined and investigated posting trends, content, and characteristics of the posts [5,16,20,21]. More than 1600 original posts were collected, and 20.30% of the posts were recognized as misleading messages during the outbreak.

### Posting Trend of the Topic

The weekly posting trend of the topic was consistent with the change of weekly reported cases in our study. From the beginning of the outbreak, microbloggers tend to rush to social media to seek information about the disease [18,20,22]. Relevant posts had occurred accompanying microbloggers’ queries on the safety of the avian-origin food. However, the number of posts about this topic did not increase much in the first 2 weeks. With the increment of reported cases, people are likely to perceive more risk and severity [23-25]. In addition, limited information sources and unanswered questions could also raise people’s anxiety and help generate misconceptions about the disease [23,26]. Accompanying the sharply rising number of reported cases at the beginning of week 3, misleading messages occurred on April 15, and the posting trend was greatly boosted from then on. This result indicated that early intervention with clear and sense-making messages was necessary for people’s appropriate response to the outbreak during this period [16,27].

The situation reversed on April 21, 2013 when the posts under “refuting messages” started to prevail over those under misleading messages. From then on, the daily number of misleading posts decreased and was virtually controlled after April 24. Social media has the ability to purify itself and should be used as an effective information communication platform [11,28,29]. In our study, the microbloggers’ timely and effective engagement in correcting the misinformation played an important role in the self-purification of social media [18,30].

### Characteristics of the Relevant Posts Under Different Categories

In our study, the posts under misleading messages tended to receive most retweets among the 4 types of posts, suggesting their high virality on social media. The virality of the posts could be measured by the times they are forwarded, replied, and/or endorsed [22,31]. In contrast, the microbloggers of “misleading messages” had fewer followers and previous posts than other microbloggers. In other words, the microbloggers who posted misinformation seemed to be inactive on social media but their misleading messages were more likely to be disseminated. The microbloggers who posted “refuting messages” were recognized as active on social media because they had most followers and previous posts among the 4 groups. However, their correcting posts received fewer reposts as well as the least reposts. This fact suggests the need to empower these kinds of messages to strengthen the self-purification function of social media [11,28,29]. The queries and other messages received more comments than the other 2 groups. These 2 groups of posts contained more content of questions and jokes, which we think were more interactive [31]. Thus, they received more replies than the other 2 groups of posts.

In terms of the geographic distribution of the misleading posts, we found misleading posts occurred more frequently in the provinces with no H7N9 cases reported than in the provinces with cases reported. Currently, people who were physically located far from disease outbreaks could obtain information rapidly from the Internet and contribute to the circulation of misinformation [11,32]. This was different from the traditional rumor dissemination. For the nonoutbreak areas, information monitoring and communication on social media are also very important.

### Features of the Most Popular Misleading Messages

Previous studies showed that the individuals who created and/or disseminated misinformation might have the motives to draw more public attention [7,33]. Social media could be a good tool to generate such misleading but attractive information [6,9,10]. Most of the top 5 misleading posts disseminated false information that H7N9 cases had occurred in the local area because of eating “chicken feet with pickled pepper.” They told a story containing information of where, when and who. Such posts were narrative and seemed to be real. This feature was similar to the traditional rumors. However, they were short and visible on the Internet, leading to the quick dissemination because of the high interactivity of social media. These kinds of misleading posts should be clarified as a priority.

### Limitations

There are several limitations in our study. Instead of retrieving data at the beginning of the outbreak, we did it after the outbreak occurred, when some microbloggers might have deleted their posts. This might have led to information loss. The real-time data retrieval would have been preferable in future studies. Although the misinformation of the specific topic we studied was prevailing during the outbreak, it could not represent all misinformation that occurred in the same period and it may not be applied to other public health emergency. Due to the limited research resources and insufficient data, we could not conduct more secondary content analysis and observe the networking of the posts, which could have further revealed the model of the misinformation dissemination on social media.

### Conclusions

This study has some implications for public health practice and health communication on social media during disease outbreaks. We think it is important to detect the microbloggers’ common concern from the very beginning of the outbreak. It was also found necessary to release correct information in response to a misunderstood topic. The microbloggers on social media can and should be empowered to clarify wrong information by themselves. The microbloggers of posts under “misleading messages” in our study seemed to be less active, but their posts drew greater attention on social media. In the times of Web 2.0, it is of equal importance to monitor outbreak and nonoutbreak areas and prevent misinformation from spreading on social media.
